# Supplementary material for: Content-rich biological network constructed by mining PubMed abstracts
Source: BMC Bioinformatics. 2004 Oct 8;5:147. doi: 10.1186/1471-2105-5-147 (PMC528731; doi:10.1186/1471-2105-5-147)
Supplement: Additional File 2 — The original results of the above study (non-essential files are deleted to keep the file size under the limit set by BMC bioinformatics). [file 1471-2105-5-147-S2.bz2 › chilibotAdditionalFile2/dip05/769ID10633096E18160/html/FECI_FECR.html]

 


 **FECI** and **FECR** 
  
Found 19 abstracts in PubMed, retrieved 05.  
 

 What does Google say? 
 PDF only 
| .edu only 

---

**Interactive relationship** (e.g. stimulation, inhibition, etc)

**Neutral relationship**- The results demonstrate that region 4 of  **FecI**  specifically binds  **FecR** .  Ref: 12057967 J Bacteriol, 2002
- no interactions occurred between heterologous combinations of the P. aeruginosa proteins and between a P. aeruginosa  **FecI**  homolog and E. coli  **FecR** .  Ref: 12057967 J Bacteriol, 2002
- Interaction of N proximal regions of predicted  **FecR**  homologs with region 4 of predicted  **FecI**  homologs of Pseudomonas aeruginosa was demonstrated.  Ref: 12057967 J Bacteriol, 2002
- An in vivo deletion analysis using a bacterial two hybrid system assigned the interaction of the  **FecR**  and  **FecI**  proteins to the cytoplasmic portion of the  **FecR**  transmembrane protein and region 4 of  **FecI** .  Ref: 12057967 J Bacteriol, 2002
- Functional interaction of region 4 of the extracytoplasmic function sigma factor  **FecI**  with the cytoplasmic portion of the  **FecR**  transmembrane protein of the Escherichia coli ferric citrate transport system.  Ref: 12057967 J Bacteriol, 2002
- **FecR**  then transmits the signal across the cytoplasmic membrane into the cytoplasm and activates the  **FecI**  sigma factor, which binds to the RNA polymerase core enzyme and directs the RNA polymerase to the promoter upstream of the fecABCDE transport genes to initiate transcription.  Ref: 12732308 Curr Opin Microbiol, 2003
- **FecR**  receives the signal in the periplasm from the outer membrane protein FecA loaded with ferric citrate, transmits the signal across the cytoplasmic membrane, and converts  **FecI**  in the cytoplasm to an active sigma factor.  Ref: 12813067 J Bacteriol, 2003
- Interaction of  **FecR**  with  **FecI**  was demonstrated by showing that isolated  **FecR**  inhibited degradation of  **FecI**  by trypsin.  Ref: 12618442 J Bacteriol, 2003
- Binding of  **FecI**  to beta and beta 1 313 was enhanced by  **FecR**  1 85, which represents the cytoplasmic portion of the  **FecR**  protein that transmits the inducing signal across the cytoplasmic membrane.  Ref: 12618442 J Bacteriol, 2003

**Non-interactive relationship** (e.g. studied together, co-existance, homology, etc.)

- Missense mutations randomly generated by PCR were localized to region 4 of  **FecI** , and the mutants were impaired with regard to the interaction of  **FecR**  with  **FecI**  and fecB lacZ transcription.  Ref: 12057967 J Bacteriol, 2002
- Therefore, transcription of the fec transport genes is subjected to double control cells first detect iron deficiency and respond by synthesis of the regulatory proteins  **FecI**  and  **FecR** , which initiate transcription of the fec transport genes, provided ferric citrate is available.  Ref: 12732308 Curr Opin Microbiol, 2003
- This binding elicits a signal that is transmitted by  **FecR**  across the cytoplasmic membrane into the cytoplasm, where the sigma factor  **FecI**  directs the RNA polymerase to the promoter upstream of the fecABCDE genes.  Ref: 12057967 J Bacteriol, 2002
- Five  **fecI**   **fecR**  iron receptor gene clusters from P. putida were also identified in the human pathogen Pseudomonas aeruginosa.  Ref: 12534467 Environ Microbiol, 2002
- In 11 cases, a  **fecR**  like gene was found adjacent to the  **fecI**  like gene and, in 10 cases, a gene encoding an iron receptor lies in the vicinity of the  **fecI**   **fecR**  cluster.  Ref: 12534467 Environ Microbiol, 2002
- Three specific proteins are involved FecA in the outer membrane,  **FecR**  in the cytoplasmic membrane, and  **FecI**  in the cytoplasm.  Ref: 12732308 Curr Opin Microbiol, 2003
